# The accuracy of the Candida Score^®^ in predicting the likelihood of fungal sepsis in newborns

**DOI:** 10.1080/07853890.2025.2548022

**Published:** 2025-08-22

**Authors:** Johanes Edy Siswanto, Milda, Dewi Patriani, Boromeus Abyasa Daniswara, Christophorus Adi S. Pamungkas

**Affiliations:** ^a^ Harapan Kita National Women and Children Health Centre, Jakarta, Indonesia; ^b^ Faculty of Medicine, Pelita Harapan University, Tangerang, Indonesia; ^c^ Mokopido Regional Public Hospital, Tolitoli, Central Sulawesi, Indonesia; ^d^ Dr. Soekardjo Regional Public Hospital, Tasikmalaya, West Java, Indonesia; ^e^ Faculty of Medicine, Gajah Mada University, Special Region Yogyakarta, Indonesia; ^f^ Faculty of Medicine, University of Indonesia, Depok, Indonesia

**Keywords:** Neonatal candidemia, Revised Candida Score^®^, fungal sepsis, thrombocytopenia, risk prediction

## Abstract

**Background:**

Candidemia poses a significant health challenge in neonates. This study evaluates a modified version of the Candida Score to enhance early detection and guide antifungal therapy decisions.

**Objective:**

To assess the accuracy of a revised Candida Score^®^ that integrates thrombocytopenia and patient origin into the original parameters.

**Methods:**

A retrospective case-control study was conducted at the Harapan Kita National Women and Children Health Centre (HKNWCHC) from 2017 to 2023. The study involved 32 neonates diagnosed with candidemia and 29 with bacterial sepsis. The original Candida Score – comprising total parenteral nutrition (TPN), surgery, multifocal colonisation, and severe sepsis – was modified by adding platelet count and patient origin. Multivariate logistic regression identified predictive factors, while ROC curve analysis validated the revised scoring system.

**Results:**

Severe thrombocytopenia (AOR 7.153; *p* = 0.043) and outborn status (AOR 6.035; *p* = 0.014) were significantly associated with candidemia. The revised Candida Score^®^ showed sensitivity of 81.3%, specificity of 58.6%, positive predictive value (PPV) of 68.4%, negative predictive value (NPV) of 62.9%, and an area under the curve (AUC) of 0.743 (*p* = 0.001).

**Conclusion:**

Incorporating outborn status and thrombocytopenia improved early identification of neonatal candidemia. The revised Candida Score^®^ is a practical tool for empirical antifungal guidance in resource-limited settings. Its high sensitivity makes it an effective screening tool, despite moderate specificity.

## Introduction

Severe neonatal infections, particularly sepsis, affect over half a million newborns globally [1,2]. Fungal sepsis, though less common than bacterial sepsis, has high mortality (19–36%) and morbidity (57.2%) rates [3,4]. Invasive fungal infections (IFI) in neonates have significantly decreased, according to a survey of 322 neonatal intensive care units (NICUs) conducted over 14 years. The number of cases per 1000 births decreased from 3.6 to 1.4. Infants weighing less than 750 g at birth showed the most significant decline, with rates falling from 82.7 to 23.8 per 1000. These infections have a 30% chance of killing premature and low-birth-weight (LBW) infants and can cause significant health problems [5]. ELBW neonates face a higher risk of disseminated Candida infections, with reported rates as high as 20%. This prevalence varies by gestational age. A study by Zhou et al. of 26 neonatal intensive care units (NICUs) in Canada found that invasive candidiasis increases with decreasing gestational age [5–7]. Factors contributing to fungal sepsis include prematurity, very low birth weight, vascular access devices, parenteral feeding, and extended hospital stays [8,9]. Although Candida species are the most commonly identified agents, diagnosing fungal sepsis is challenging, and blood culture is still the primary diagnostic for verifying the diagnosis [10]. Thrombocytopenia, defined as a platelet count <150,000/mm³, has emerged as a potential early indicator – especially in fungal sepsis – yet neonatal-specific evidence remains limited [11–13].

León et al. originally proposed the Candida Score to assist in distinguishing colonization from invasive candidiasis among critically ill adult ICU patients without neutropenia. The score was based on clinical indicators, including total parenteral nutrition, surgical intervention, multifocal colonization, and severe sepsis. However, its applicability in neonates, who differ substantially in physiology and clinical presentation, remains uncertain [14,15]. Therefore, this study aims to modify and validate the existing Candida Score for use in neonatal populations by adjusting its components to better reflect neonatal clinical characteristics and support timely antifungal decision-making.

## Materials and methods

### Study design

This retrospective case-control study was conducted at the Harapan Kita National Women and Children Health Centre (HKNWCHC) in Jakarta, Indonesia, over the period from 2017 to 2023. The research received ethical approval from the institutional review board (IRB/34/06/ETIK/2024). All procedures performed in studies involving human participants were in accordance with the ethical standards of the institutional research committee and with the 1964 Helsinki Declaration and its later amendments or comparable ethical standards.

### Participants

The study included a total of 61 neonates presenting clinical signs of sepsis. Among these, 32 were diagnosed with culture-confirmed candidemia, while 29 were identified with bacterial sepsis. Data were extracted from anonymized medical records.

### Candida Score modification

The original Candida Score consists of four components: total parenteral nutrition, surgery, multifocal colonization, and severe sepsis, with each component contributing one point. A total score of ≥3 suggests a high risk for invasive candidiasis. In this study, the score was enhanced by incorporating two additional variables identified through logistic regression analysis and scoring algorithm: (1) patient origin (inborn vs. outborn) and (2) severity of platelet count (normal, mild, or severe thrombocytopenia). Regression coefficients were converted into point values using a beta coefficient scaling approach to develop a revised Candida Score^®^.

### Statistical analysis

Bivariate analyses were conducted to identify candidate predictors (*p* < 0.25), which were subsequently entered into a multivariate logistic regression model. The model’s performance was evaluated using receiver operating characteristic (ROC) curve analysis, incorporating metrics such as area under the curve (AUC), sensitivity, specificity, positive predictive value (PPV), negative predictive value (NPV), and kappa statistics.

## Results

During the period from 1 January 2017 to 31 December 2023, we identified 32 patients with positive fungal cultures. Unfortunately, we experienced a 42% overall mortality rate, with 15 infants succumbing to the condition. As part of our research, we gathered 61 patient records that were diagnosed with sepsis, 32 of which had candidemia. The remaining 29 patients were included as a control group. Table 1 contains detailed patient data and bivariate analysis between groups.

**Table 1.** Characteristics of research subjects.

Characteristics	Neonatal Sepsis		*p*-Value
	Fungal Sepsis (*n* = 32)	Bacterial Sepsis (*n* = 29)	
Sex, *n* (%)			0.395^1^
Male	20 (62.5)	15 (51.7)	
Female	12 (37.5)	14 (48.3)	
Birth weight (gram)			0.855^2^
Mean (95% CI)	1868 (1596–2133)	1907 (1575–2217)	
Gestational age (weeks)			0.872^3^
Median (range)	34 (26–37)	34 (26–38)	
Classification			0.819^1^
Appropriate for gestational age	24 (75.0)	21 (72.4)	
Small for gestational age	8 (25.0)	8 (27.6)	
Patient origin			0.055^1^
Inborn infants	12 (37.5)	18 (62.1)	
Outborn infants	20 (62.5)	11 (37.9)	
Duration of parenteral nutrition (days)			0.811^3^
Median (range)	13.,5 (1–65)	17,0 (3–97)	
Length of stay (days)			0.026^3^
Median (range)	30 (5–95)	42 (10–97)	
Hemoglobin level (mg/dL)			0.086^2^
Mean (95% CI)	12,0 (11.2–12.9)	13,3 (12.1–14.5)	
Platelet counts (/mm3)			0.051^3^
Median (range)	93.000 (7.000–460.000)	150.000 (11.000–562.000)	
Monocyte counts (/mm3)			0.934^2^
Mean (95% CI)	13.4 (11.4–15.3)	13,5 (10.7–16.7)	
Severe Sepsis			0.065^1^
Yes	15 (46.9)	7 (24.1)	
No	17 (53.1)	22 (75.9)	
Candida score			
Median (range)	2,5 (1–5)	1 (1–4)	0.005^3^
Operative procedures, *n* (%)			0.107^1^
Yes	14 (43.8)	7 (24.1)	
No	18 (56.2)	22 (75.9)	
Mechanical ventilation, *n* (%)			0.059
Yes	24 (75.0)	15 (51.7)	
No	8 (25.0)	14 (48.3)	

Analyzed using Chi-square test(^1^), independent-*t* test(^2^), and Mann-Whitney *U* test(^3^).

**Figure 1.**
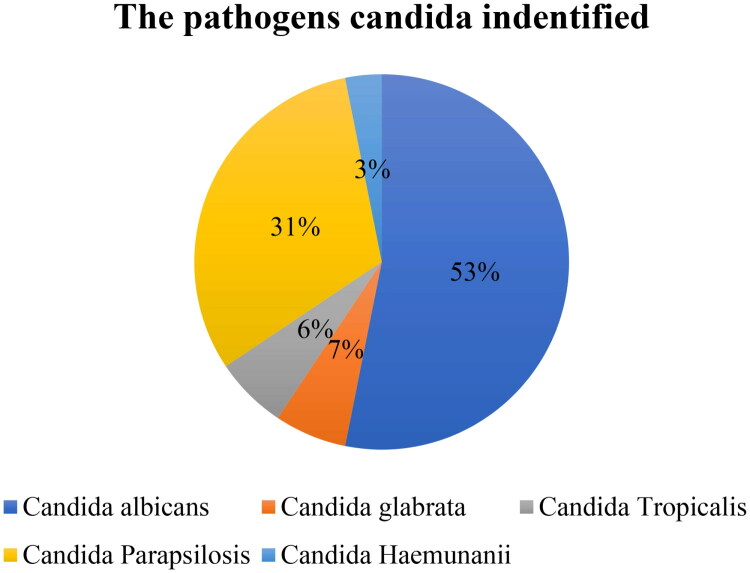
Distribution of types of Candida pathogens identified in blood culture results.

The study was conducted on 61 neonates, among whom 32 were diagnosed with fungal sepsis and 29 with bacterial sepsis. The pathogens identified were *Candida albicans* (53%) and *Candida non-albicans* (46.8%), including *Candida glabrata* (6.25%), *Candida tropicalis* (6.25%), *Candida parapsilosis* (31.25%), and *Candida haemulonii* (3.1%). The mortality rate was higher for *Candida albicans* (53.3%) compared to *Candida non-albicans* (41.2%). (Figure 1) Most demographic and clinical characteristics were not significantly different, with the exception of patient origin, platelet levels, the current candida score, mechanical ventilator, and length of stay in the NICU, which were statistically significant (Table 1).

Through multivariate logistic regression analysis (Table 2), this study identified two novel independent risk factors for neonatal candidemia: outborn status (AOR 6.035; *p* = 0.014) and severe thrombocytopenia (<50,000/mm³) (AOR 7.153; *p* = 0.043). These variables were not included in the original Candida Score^®^, which in our cohort, did not demonstrate a statistically significant association with candidemia risk. To enhance predictive accuracy, we integrated these two factors into the existing scoring framework. The revised model, based on simplified scoring derived from regression coefficients (Table 3) [16], demonstrated superior performance, with an ROC AUC of 0.743, indicating improved discriminative ability.

**Table 2.** Multivariate logistic regression of risk Factors associated with candidemia.

Variable	Crude Odds Ratio (CI 95%)	*p*-Value	Adjusted Odds Ratio (CI 95%)	*p*-Value
**Gender**				
Female	1			
Male	1.556 (0.560–4,319)	0.396		
**Candida score**				
< 3	1	***0.041***	1	0.275
≥ 3	3.143 (1.049–9,414)		2.444 (0.490–12.178)	
**Platelet counts**				
Normal	1		1	0.333
Mild-moderate	1.515 (0.461–4.981)	0.494	2.095 (0.469–9.367)	***0.043***
Severe	7.222 (1.607–32.464)	***0.010***	7.153 (1.060–48.271)	
**Monocytosis**				
No	1			
Yes	1.607 (0.509–5.073)	0.419		
**Anemia**				
No	1		1	0.397
Yes	2.008 (0.631–6.388)	0.238	1.785 (0.467–6.819)	
**Mechanical ventilator**				
No	1		1	0.712
Yes	2.800 (0.949–8.262)	0.062	1.344 (0.279–6.468)	
**Patient origin**				
Inborn infants	1		1	***0.014***
Outborn infants	2.727 (0.967–7.691)	0.058	6.035 (1.438–25.340)	
**Classification**	1			
AGA				
SGA	0.875 (0.279–2.740)	0.819		
**Birth weight**	1			
≥ 1500 gram				
< 1500 gram	0.744 (0.258–2.145)	0.584		
Gestational age	1			
≥ 32 weeks				
< 32 weeks	1.010 (0.341–2.990)	0.986		

AGA: appropriate for gestational age; SGA: small for gestational age.

**Table 3.** Modified calculation of scores for *Candida Score^®^.*

	AOR (95% CI)	*p*-Value	*B*	B × 10 rounded	*β*/smallest number	Scoring model
Candida score						
< 3	1	0.275	–	–	−1.3	0
≥ 3	2.4 (0.5–12.2)		1.2	12		1.3
Platelet count						
Normal	1		–	–	–	0
Mild-moderate	2.1 (0.5–9.4)	0.333	0.9	9	1	1
Thrombocytopenia Severe thrombocytopenia	7.2 (1.1–48.3)	***0.043***	4.1	41	4.6	4.6
Patient origin						
Inborn infants	1		–	–	–	0
Outborn infants	6.0 (1.4–25.3)	***0.014***	6.0	60	6.7	6.7

*Candida Score^®^: Revised Candida Score*.

Table 4 evaluated the test’s accuracy using the ROC curve. The candidemia score was validated by comparing it to the study and control groups, using a cut-off value of >5.25, as shown in Table 4. The revised Candida Score^®^, which included these two variables in addition to the original four (TPN, surgery, multifocal colonization, and severe sepsis), showed improved diagnostic accuracy. The model yielded a sensitivity of 81.3%, specificity of 58.6%, PPV of 68.4%, NPV of 62.9%, and an area under the ROC curve (AUC) of 0.743 (*p* = 0.001). These findings indicate that the scoring was a reliable and accurate diagnostic tool. The combination of candida score, severe thrombocytopenia, and patient origin proved to be effective in differentiating patients with and without candidemia.

**Table 4.** Diagnostic performance of the revised Candida Score^®^.

	Candidemia diagnostic model	
	Probabilistic models	Candida score^®^ Model
Formula	Logit (Y | Candida score) = candida score * 0.894 +	*β* coefficient × 10
	mild Tr * 0.740 + severe Tr * 1.968 + patient origin	The results of the smallest (*β* × 10)
	* 1.798 (*p*-value = 0.009).	
		First, multiply *β* by 10. Then, divide the results by the smallest number from step 1.
ROC Curve	**AUC (95% CI): 0.743 (0.619 − 0.867)**	**AUC (95% CI): 0.743 (0.619 − 0.867)**
	*p*-Value: 0.001	*p*-Value: 0.001
	Cut-off point > 5.25	Cut-off point > 5.25
Diagnosis value	**Sensitivity:** 81,3%	**Sensitivity:** 81,3%
	**Specificity:** 58,6%	**Specificity:** 58,6%
	**PPV:** 68,4%	**PPV:** 68,4%
	**NPV:** 62,9%	**NPV:** 62,9%
	**LR (+):** 1,96	**LR (+):** 1,96
	**LR (-):** 0,32	**LR (−):** 0,32
**Kappa score**	**100%**	

In the subsequent stage of our research, we conducted an internal validation on 32 cases to compare the effectiveness of the probabilistic and Candida scores with the Candida culture results, which are considered the definitive standard. We employed Kappa values as a metric to evaluate the dependability of both models in forecasting the likelihood of candida infection. Kappa statistics are commonly used to test the consistency of evaluations. After analyzing the AUC and Kappa values, we arrived at the conclusion that the revised Candida score is a reliable tool to assess and predict the incidence of neonatal candidemia (Table 4.)

Table 5. Below is the final model for Candida’s score with a cut point of 5.25. If the calculation results exceed the limit values, the risk of candida infection arises.

**Table 5.** Model risk prediction model for neonatal candidemia based on the revised Candida Score^®^ (Final Scoring Model).

Variable	Xn (Choose one below)	Score	Ʃ (sum of model scores)
Existing Candida Score			
Severe sepsis			(X_1_+ X_2_ + X_3_+X_4_) ≥ 3 (X_1_+ X_2_ + X_3_+X_4_) < 3
Yes	2	X_1_	
No	0		
Total parenteral nutrition		X_2_	
Yes	1		
No	0		
Multifocal colonization			
Yes	1	X_3_	
No	0		
Surgery			
Yes	1	X_4_	
No	0		
Candida Score^®^			
Existing Candida score			(Y_1_ + Y_2_ + Y_3_)
*X* ≥ 3	1.3	Y_1_	
*X* < 3	0		
Platelet count			
Severe thrombocytopenia	4.6		
Mild thrombocytopenia	1	Y_2_	
Normal	0		
Patient origin			
Outborn infants	6.7	Y_3_	
Inborn infants	0		

*Note.* Scoring Model A Candida sepsis if the sum of Candida scores^®^: (Y_1_ + Y_2_ + Y_3_) > 5.25.

## Discussion

Many new ideas for reducing the risk of bacterial and fungal sepsis are being developed. Notably, Li et al.’s (2022) findings demonstrate the clinical relevance and comparative superiority of the revised Candida score model, which adheres to the Sepsis 3.0 definition, over the prior sepsis/severe sepsis model in assessing critically ill patients newly admitted to the ICU [17]. Predicting mortality scores will be beneficial for a similar investigation. A case-control study that contrasts IFI with the control is a worthwhile solution to develop [18]. We conducted a review of 32 infants with Candida infections, finding a 1.6:1 male-to-female ratio [19–24]. Our analysis did not reveal any significant correlation between gestational age, birth weight, gender, anemia, monocytosis, or mechanical ventilation. However, we observed lower birth weights in cases of persistent candidemia, which is consistent with the findings from Fu’s study [25]. Omima’s 2020 research highlighted catheterization, total parenteral nutrition, mechanical ventilation, prolonged antibiotic use, and extended hospital stays as significant factors [26], supporting Wadile and Bhate’s observations on invasive devices spreading pathogens [27].

We discovered five Candida species: *C. albicans*, *C. parapsilosis complex*, *C. tropicalis*, *C. glabrata complex*, and *C. haemulonii*, with *C. albicans* being the most common (53%). This finding is consistent with Hassan and Jain’s [28,29] studies, although the prevalence order varies by region, with non-albicans species being more prevalent in Asia [28]. Additionally, Auriti et al. found a 70% increase in *C. albicans* vaginal candida colonization during pregnancy, contributing to obstetric tears [30].

Critically ill individuals, especially those experiencing multi-organ dysfunction or advanced-stage illness, are particularly vulnerable to developing candidemia. Our research involved observing 32 infants, of whom 15 sadly passed away, resulting in a 42% mortality rate. The global newborn invasive candidiasis (NIC) death rate ranges from 12% to 37% in high-income countries (HICs) to 8.9–75% in low- and middle-income countries (LMIC). Our investigation indicates mortality rates of 53.3% for C. albicans and 41.2% for non-albicans, which are similar to Gaffari Tunc’s findings [31,32].

Platelets are crucial in protecting the body against infections. Our study has found that a significant decrease in platelet count below 50,000 may indicate fungal sepsis in newborns and serve as a potential detection marker. According to studies by James M. et al. and Calaveras TS et al. 70% of newborns with fungal sepsis experience thrombocytopenia [33,34].Parvez A. et al. reported greater mean platelet counts at the onset of sepsis in gram-negative and fungal sepsis [35], but Guida JD et al. reported considerably lower platelet counts in these instances [36]^6^. Platelets link innate and adaptive immune responses [36]. In septic newborns, thrombocytopenia can be caused by platelet destruction, reduced production, or a combination of endothelial damage, microbial toxins, and DIC [37]^7^. Platelets, namely TLR4, play a crucial role in connecting innate and adaptive immune responses [38,39].

According to our data, the risk of candidemia appears to be higher among outborn neonates – those delivered outside the referral hospital setting. Neonates delivered outside tertiary care hospitals are frequently referred from under-resourced facilities, where limitations in early sepsis recognition, prompt initiation of empirical therapy, and implementation of infection control protocols may lead to delayed and inadequate management. Such conditions increase the likelihood of early exposure to broad-spectrum antibiotics, invasive procedures, or suboptimal care during transport, all of which are known contributors to invasive fungal infections [30,31]. Inadequate antimicrobial stewardship and limited access to antifungal susceptibility testing in referring centers may further compromise timely diagnosis and treatment. These findings highlight the importance of incorporating non-physiological but contextually relevant factors – such as place of birth – into risk prediction models. In our cohort, the current Candida Score may be insufficient to fully capture the risk of systemic candidiasis, although a score greater than three still identified high-risk patients (OR 3.143, *p* > 0.275). Lambiotte et al. have similarly shown increased antifungal use in patients with elevated Candida scores [8,40].

Several studies cited in this discussion, including those by León et al. [14], Ostrosky-Zeichner and Pappas [41], Tissot et al. [42], and Ratridewi et al. [43], were developed in the context of adult or immunocompromised populations, such as non-neutropenic ICU patients or cancer patients with severe neutropenia. These populations differ substantially from neonates in both physiology and clinical presentation. In contrast, our study specifically focuses on high-risk neonates admitted to the NICU, highlighting the urgent need for neonatal-specific adaptations of existing risk prediction tools. Unlike the original Candida Score, which aimed to differentiate colonization from invasive candidiasis in adults, the revised score in our study is designed to distinguish between fungal and bacterial sepsis in neonates presenting with clinical signs of infection. This distinction highlights a more significant diagnostic challenge encountered in neonatal intensive care, underscoring the importance of tailored scoring models for this vulnerable population. The revised Candida Score^®^ demonstrates a sensitivity of 81%, specificity of 59%, positive predictive value (PPV) of 68%, and negative predictive value (NPV) of 63%. A score exceeding 5.25, along with the presence of severe thrombocytopenia and outborn status, may serve as a practical indicator for initiating early empirical antifungal therapy [8,11,41–43].

These findings highlight the practical clinical utility of the revised Candida Score^®^ as a bedside tool for predicting candidemia in neonates with clinical sepsis. By integrating two routinely available parameters – patient origin and platelet count severity – into the existing scoring system, clinicians can better stratify risk and identify neonates who may benefit from early empirical antifungal therapy. This is particularly relevant in low-resource settings, where delays in blood culture results or limited access to fungal biomarkers (e.g. β-D-glucan, mannan antigen) can hinder timely diagnosis. The simplicity of the score and its reliance on standard clinical data make it feasible for implementation in both tertiary and district-level neonatal care units, and potentially valuable for integration into neonatal sepsis protocols to reduce treatment delays and improve outcomes. Given the urgency of timely diagnosis in neonatal candidemia, a high sensitivity is more desirable than specificity in this context. Although the revised score shows only moderate specificity (58.6%), its strong sensitivity (81.3%) makes it an effective early screening tool to flag high-risk neonates for further evaluation or empirical antifungal therapy.

## Limitations

This study has several limitations. First, the relatively small sample size may affect the generalisability of the findings and limit the statistical power of subgroup analyses. Second, the retrospective design may be subject to information bias and relies on the completeness of medical records. Third, the model was developed and validated using data from a single tertiary centre, which may restrict its applicability to other settings. Future studies with larger, multicentre cohorts are needed to externally validate the revised Candida Score^®^ and assess its performance across diverse neonatal populations.

## Conclusion and policy implication

The revised Candida Score^®^ offers a practical and moderately accurate approach for the early identification of candidemia in neonates with clinical sepsis. For ease of application in daily practice, the revised score may be adapted into a simplified bedside chart or digital calculator. While it is not intended to replace microbiological confirmation, it provides valuable guidance for initiating empirical antifungal therapy, especially in high-risk infants and settings with limited diagnostic resources. A score above 5.25, as validated through ROC analysis, may serve as a trigger point for early intervention, potentially improving survival outcomes by reducing delays in treatment.

Integration of this scoring system into neonatal sepsis protocols could support more timely and targeted clinical decision-making. In low-resource settings, its reliance on routinely collected clinical data enhances feasibility for widespread adoption. To maximise its effectiveness, hospitals should consider forming multidisciplinary antifungal stewardship teams and adopting national or institutional guidelines that include this scoring tool. Such measures may contribute to improved diagnostic accuracy, more rational antifungal use, and a reduction in fungal-related morbidity and mortality among vulnerable newborns.

## Data Availability

This research utilizes physical samples that are not available for direct sharing. However, detailed data can be provided upon request from the corresponding author, ensuring access to vital information while maintaining the integrity of the samples.
